# A Review of Robotic Weeding Modalities for Site-Specific Weed Management

**DOI:** 10.3390/s26102925

**Published:** 2026-05-07

**Authors:** Feng Gao, Shugui Ding, Wenpeng Zhu, Kang Han, Bin Wu, Maocheng Zhao, Zhong Li, Xiaojun Jin

**Affiliations:** 1 Agricultural and Forestry Products Deep Processing Technology and Equipment Engineering Center of Jiangsu Province, Nanjing Forestry University, Nanjing 210037, China; 2 National Engineering Research Center of Biomaterials, Nanjing Forestry University, Nanjing 210037, China; 3 Nanjing Lehui Finnah Packtec Co., Ltd., Nanjing 211151, China; 4 Center for Agricultural Robotics and Automation, Jurong Institute of Smart Agriculture, Zhenjiang 212441, China; 5 College of Mechanical and Electronic Engineering, Nanjing Forestry University, Nanjing 210037, China

**Keywords:** robotic weeding, site-specific weed management, weeding modalities, sensing–actuation coupling, precision agriculture

## Abstract

Weed control remains a critical challenge in modern crop production, particularly under increasing pressure to reduce chemical inputs and improve environmental sustainability. Recent advances in precision agriculture and robotic systems have enabled site-specific weed management, where interventions are applied selectively based on detected weed locations. While extensive research has focused on improving weed detection algorithms, comparatively less attention has been paid to the characteristics and constraints of different weeding modalities, which ultimately determine field performance. This review presents a systematic analysis of robotic weeding modalities from an actuation-oriented perspective. Specifically, we establish a comprehensive taxonomy of weeding approaches, including mechanical, chemical, thermal, laser-based, electrical, and other emerging methods, and analyze their underlying mechanisms and operational characteristics. Furthermore, we examine the coupling between sensing and actuation, highlighting how different intervention modalities impose distinct requirements on perception outputs. A scenario-based comparison framework is then developed to evaluate the suitability of different modalities across representative agricultural conditions, including pre-emergence control, in-row selective weeding, dense-row crop systems, and large weed situations. Based on this analysis, the limitations of single-modality systems are discussed, and emerging trends toward multi-modality integration and air–ground collaborative weed management are reviewed. Overall, this review shifts the focus from detection-centric approaches to the integration of sensing and actuation in robotic weeding systems and provides a decision-oriented framework to support the design, selection, and deployment of next-generation robotic weed management technologies.

## 1. Introduction

Weeds remain one of the most persistent biological constraints in crop production, competing with cultivated plants for water, nutrients, light, and space, and leading to significant yield and quality losses worldwide [1]. Conventional weed management has long relied on broadcast herbicide application and mechanical cultivation [2]. However, increasing herbicide resistance, environmental contamination, regulatory restrictions, and growing societal demand for sustainable agriculture have exposed the limitations of uniform, field-wide treatments [3,4]. In this context, weed control is no longer merely an agronomic operation but has become a central issue in sustainable crop production systems, requiring approaches that reduce chemical input while maintaining high levels of efficacy and operational efficiency [5].

The rapid advancement of precision agriculture technologies has enabled a shift from broadcast weed management to site-specific weed management, where interventions are applied only to detected weed targets [6,7]. Developments in machine vision, deep learning, real-time positioning systems, and autonomous agricultural platforms have facilitated increasingly accurate weed recognition under field conditions [8,9,10]. As a result, robotic weeding systems have emerged as a promising pathway toward reducing chemical inputs and enabling selective interventions at the plant scale [6,7,11]. However, while recognition performance has improved substantially in recent years, effective weed control in real-world conditions depends not only on detection accuracy but also on the characteristics and constraints of the chosen intervention modality [12].

A large body of recent review literature has primarily focused on weed detection algorithms, particularly deep learning-based classification and segmentation methods [13]. These studies systematically evaluate detection performance using metrics such as precision, recall, and mean average precision and provide valuable insights into dataset design and model architecture optimization [14]. Nevertheless, detection accuracy alone does not guarantee successful weed suppression. Different intervention modalities—mechanical removal, chemical microdosing, thermal treatment, laser ablation, or electrical destruction—impose distinct requirements on spatial accuracy, timing, energy delivery, and crop safety [7,15]. Moreover, factors such as soil conditions, weed growth stage, perennial root systems, actuator throughput, and operational safety strongly influence field performance [7]. Therefore, weed detection and physical intervention should be considered a tightly coupled system rather than independent components [13,16].

Despite the growing number of robotic weeding studies, comparatively fewer reviews have systematically examined the engineering characteristics and application boundaries of different weeding modalities from an actuation-oriented perspective [7,17]. This review addresses this gap by focusing on robotic weeding modalities within site-specific weed management systems [6]. Specifically, this paper (1) establishes a unified taxonomy of weeding modalities, including mechanical, chemical, thermal, laser-based, electrical, and other emerging approaches; (2) analyzes their physical mechanisms and operational constraints; (3) compares modality suitability across representative field scenarios such as pre-emergence control, in-row selective weeding, dense-row crops, and large weed situations; and (4) discusses emerging system-level trends, including multi-modality integration and air–ground collaborative strategies for robotic weed management. By shifting the focus from detection algorithms alone to the detection–actuation coupling and engineering feasibility, this review aims to provide a decision-oriented reference framework for researchers and system developers [16].

To ensure comprehensive coverage, a structured literature search was conducted across Web of Science, Scopus, ScienceDirect, IEEE Xplore, SpringerLink, and Google Scholar, covering publications from approximately 2000 to 2026. Keyword combinations included “robotic weeding,” “autonomous weeding robot,” “end-effector,” “mechanical weeding,” “laser weeding,” “thermal weed control,” “electrical weed control,” “spot spraying,” and “site-specific weed management.” Journal articles and conference papers reporting experimental validation, system implementation, or detailed technical analysis were included. In addition, selected reports on commercial robotic weeding systems were considered to capture practical engineering developments. The retrieved literature was analyzed with emphasis on intervention mechanisms, actuator structures, performance constraints, and system integration rather than solely algorithmic detection metrics.

The rest of the paper is organized as follows. Section 2 presents a comprehensive taxonomy of robotic weeding modalities based on their underlying intervention mechanisms, including mechanical, chemical, thermal, laser-based, electrical, and other emerging approaches. Section 3 examines the coupling between sensing and actuation, emphasizing how different intervention modalities impose distinct requirements on perception outputs. Section 4 provides a scenario-based comparison of weeding modalities across representative agricultural conditions and develops a decision framework for modality selection. Section 5 discusses current limitations of single-modality systems and highlights emerging trends in hybrid weeding strategies and air–ground collaborative systems. Section 6 offers a critical discussion on key technological bottlenecks, trade-offs, and research gaps, followed by the conclusions in Section 7.

## 2. Robotic Weeding Modalities and Intervention Mechanisms

The effectiveness of site-specific weed management systems ultimately depends on how weeds are physically neutralized after detection [18]. Although advances in perception technologies enable increasingly accurate weed localization, the intervention modality determines the actual agronomic outcome, including weed mortality, regrowth risk, crop safety, operational efficiency, and energy consumption [19,20]. Robotic weeding systems employ a diverse range of intervention approaches that differ in energy delivery mechanisms, structural configurations, and field applicability [21,22].

**Figure 1:**
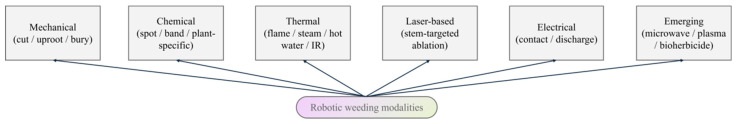
Taxonomy of robotic weeding modalities based on intervention mechanisms.

Figure 1 illustrates the overall taxonomy of robotic weeding modalities considered in this review and highlights their classification based on the underlying intervention mechanisms. To provide a systematic comparison, this section categorizes robotic weeding modalities into five primary groups based on their underlying physical mechanisms: mechanical, chemical, thermal, optical, and electrical approaches. For each modality, the fundamental working principles, operational characteristics, and typical application boundaries are discussed. This structured analysis forms the basis for subsequent cross-modality comparison and scenario-based evaluation.

### 2.1. Mechanical Weeding

Mechanical weeding was the earliest technique applied in robotic site-specific weed control. It directly destroys weed roots and growing points through physical operations such as cutting, hoeing, rotary tillage, or uprooting, thereby achieving effective weed removal or suppressing regrowth [23,24,25,26,27,28]. Compared with chemical weed control, its principal advantage is the complete elimination of herbicide-residue risk. This feature gives mechanical weeding substantial potential and practical value in organic agriculture, low- or reduced-pesticide production systems, and the cultivation of high-value horticultural crops [24,25,26,27,29].

According to their operating mechanisms, mechanical weeding systems can generally be classified into four typical modes: (1) cutting, in which blades or oscillating hoes sever weed stems at the base within the shallow soil layer; (2) uprooting, in which seedlings are pulled out by the roots using clamping, belt-driven, or flicking devices; (3) burial, in which hilling or soil covering blocks light and thereby suppresses weed growth; and (4) root disturbance, in which disruption of the shallow soil layer breaks root–soil contact and reduces weed survival [24,25,26,27,30,31].

**Figure 2:**
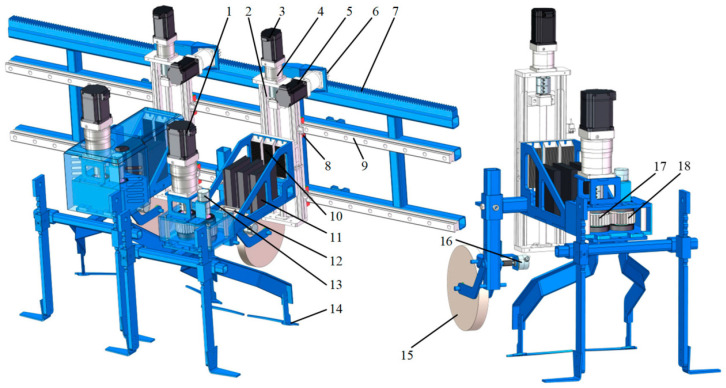
Representative structure of a smart intra-row mechanical weeding device for open-field vegetable production [36]. (1) Weeding servo motor. (2) Lifting slide table. (3) Lifting servo motor. (4) Reducer. (5) Traverse servo motor. (6) Step gear. (7) Rack. (8) Slider. (9) Slide rail. (10) Brake resistor. (11) Servo motor driver. (12) C37 controller. (13) Angle encoder. (14) Weeding knife. (15) Profiling wheel. (16) Profiling wheel angle encoder. (17) Driven gear. (18) Driven gear.

From the perspective of operational space, robotic mechanical weeding end-effectors can be divided into inter-row and intra-row configurations [24,25,26,27,32,33]. Inter-row weeding mainly targets the relatively open spaces between crop rows and typically employs rigid blades, hoes, rotary tillers, or rolling components for continuous operation. This scenario is comparatively less demanding in terms of control, with key technical considerations centered on path-tracking stability and optimization of working width for greater efficiency [24,25,26,34]. In contrast, intra-row weeding must achieve rapid crop avoidance and precise tool insertion within the confined space immediately adjacent to crop plants. Because it places extremely high demands on positional accuracy and response speed, it is widely regarded as one of the most technically challenging scenarios in mechanical weeding [24,25,26,27,35]. A representative structure of a smart intra-row mechanical weeding device is shown in Figure 2, illustrating how the actuator is arranged to operate within the narrow space between adjacent crop plants.

Early representative studies demonstrated that vision-guided mechanical intra-row weeding systems were already capable of crop recognition and synchronized blade actuation in transplanted vegetable production, thereby effectively reducing reliance on manual weeding [24,37,38]. Building on this foundation, later co-robotic systems adopted a hybrid intelligence strategy in which the more demanding perceptual correction tasks remained under human supervision, whereas the repetitive high-frequency blade insertion actions were executed by automated mechanisms. This approach not only substantially reduced labor input but also showed that mechanical end-effectors could provide meaningful labor substitution even under semi-autonomous operation [25,39].

With advances in machine vision, deep learning, and stereo matching, modern mechanical weeding robots have undergone a fundamental shift in control strategy, evolving from conventional open-loop actuation based on preset plant spacing to closed-loop control driven by real-time detection [27]. These systems identify crops and weeds in real time and dynamically generate optimal working trajectories by integrating crop safety zones, weed centroid positions, and tool motion envelopes, thereby enabling precise site-specific cutting, lateral crop avoidance, and localized soil disturbance [27]. Notably, although the introduction of deep learning has greatly improved recognition robustness in complex field backgrounds, it has also imposed new system constraints: the tolerance of the end-effector to perception latency, coordinate calibration errors, and actuator response delays is markedly reduced, and even minor errors may result in operational failure [27].

From an engineering perspective, mechanical weeding offers distinct advantages, including the absence of chemical residues, ease of modular integration, and high efficacy against larger weeds [23,24,25,26,27]. Its performance is particularly notable in near-crop operations in orchards and vineyards, where rotary cutting heads combined with adjustable positioning mechanisms have shown strong effectiveness [26].

However, mechanical weeding also has clear limitations [24,25,26,27]. First, it is highly dependent on soil conditions: excessively wet, compacted, stony, or heavily residue-covered soils can markedly reduce tool stability, weed removal efficiency, and crop safety [24,25,26]. Second, it requires extremely high control precision, often at the centimeter level or better, in both lateral positioning and temporal coordination; otherwise, missed weeds, crop injury, or repeated soil disturbance can easily occur [24,25,27]. Third, root removal is often incomplete. Although mechanical weeding is effective against weeds at the seedling stage, deep-rooted, perennial, or highly regenerative species remain prone to regrowth after a single disturbance event [23,24,27]. Fourth, operation is constrained by limited space and visibility. Under conditions of high planting density, narrow row spacing, or severe canopy occlusion, systems often face the dilemma of being able to detect weeds without sufficient access or being able to reach them without safely actuating the tool [24,25,27]. Even in orchards, operations near the trunk require actuators with complex multi-degree-of-freedom adjustment to avoid trunk collision or incomplete weed removal [26].

Overall, mechanical weeding is best suited to scenarios in which herbicide use is restricted, target plants are relatively large, precise near-crop treatment is required, and soil conditions are favorable [24,25,26,27]. To facilitate comparison of operating principles, advantages, limitations, and application contexts across different mechanical weeding mechanisms, Table 1 summarizes representative end-effectors commonly used in robotic mechanical weeding and provides supporting references for each row. The core value of mechanical weeding lies not only in replacing chemical herbicides but also in establishing a canonical perception–action closed loop of detection, localization, crop avoidance, and tool actuation [25,27]. Nevertheless, its performance ceiling is constrained by the combined trade-offs among sensing accuracy, mechanical flexibility, operating speed, and soil conditions, making further gains difficult through isolated improvements alone. Future system design will therefore likely move toward multimodal integration, in which mechanical weeding is combined with spraying, laser treatment, or electrical control technologies to achieve superior overall performance [25,27].

### 2.2. Chemical Weeding

Chemical weeding is one of the most widely used and technically mature approaches in site-specific weed management. Its core principle is to shift from conventional uniform field-wide spraying to localized, quantitative, and demand-driven herbicide application, in which intervention is guided by the spatial distribution, density, or individual positions of weeds [40,41,42,43]. Unlike conventional methods, robotic chemical weeding systems establish a complete perception–decision–execution loop: weeds are first identified using machine vision, spectral sensors, or deep learning models, and the sprayer is then actuated according to the detection results so that herbicides are applied only to target areas [40,41,42]. While maintaining weeding efficacy, this approach can substantially reduce chemical usage, lessen environmental burdens, and improve application efficiency, making it particularly suitable for fields with pronounced spatial heterogeneity in weed distribution [40,41,42,44].

**Figure 3:**
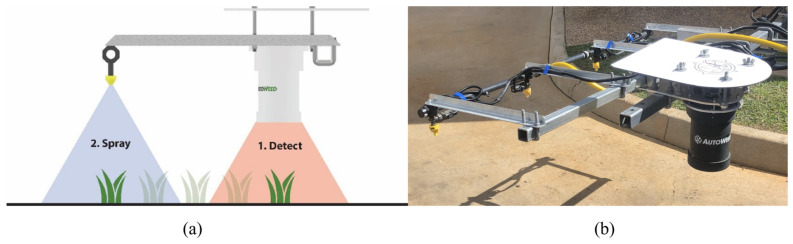
Example of a robotic spot-spraying system integrating weed detection, decision-making, and nozzle-level herbicide application [48]. (**a**) Schematic workflow showing weed detection followed by nozzle-triggered spraying. (**b**) Prototype spray boom and camera/nozzle assembly.

According to operational scale and execution mode, robotic chemical weeding can generally be classified into four main categories: patch spraying, band spraying, spot spraying, and plant-specific spraying [40,41,44]. Patch spraying mainly targets contiguous weed-infested areas and treats localized weed patches on the basis of pre-established maps or real-time detection [41,44]. Band spraying typically applies herbicides continuously along crop rows or predefined strips, providing a balance between operational efficiency and precision [41,45]. Spot spraying focuses on individual targets or small weed clusters and emphasizes rapid nozzle switching to achieve precise small-area coverage [40,44]. Plant-specific spraying represents the highest-precision mode: it requires not only weed detection but also reliable discrimination between crops and weeds, followed by precise delivery of herbicide to the effective target region of each individual weed plant [46,47]. Table 2 provides a detailed comparison of these four technical routes in terms of target scale, execution mode, and their respective advantages and limitations [40,41,44,46]. As shown in Figure 3, representative robotic spot-spraying systems integrate perception, decision-making, and nozzle-level execution into a unified platform for site-specific herbicide application.

**Table 2:** Comparison of different technical routes for robotic chemical weeding, with representative references.

Technical Route	Target Scale	Typical Execution Mode	Main Advantages	Main Limitations	Typical Application Scenarios	Representative References
Patch spraying	Area level/patch level	Spraying localized weed-infested zones based on weed distribution maps or online detection results	High coverage efficiency; suitable for large-scale operations; readily integrated with map-based management	Relatively low spatial resolution; difficult to achieve plant-level precision	Field crops; areas with patchy weed distribution	[41,44,49]
Band spraying	Row-band level	Continuous herbicide application along crop rows or predefined strip zones	Relatively simple control; balances efficiency with a certain degree of precision	Limited selectivity for scattered in-row weeds; non-target application may still occur	Row crops; regularly planted fields	[41,45,49]
Spot spraying	Single-point or small target clusters	Rapid nozzle switching to spray detected local targets	Can substantially reduce herbicide use; well suited to fields with strong spatial heterogeneity of weeds	Highly dependent on detection accuracy and nozzle response speed; requires precise timing control	Low- to medium-density weed populations; localized precision management	[40,44,48,50]
Plant-specific spraying	Individual plant level	Quantitative spraying of individual weeds after distinguishing crops from weeds	High precision; minimizes non-target contamination to the greatest extent	Imposes the highest demands on recognition, localization, calibration, and speed compensation; false detection may cause crop injury	Vegetables, high-value crops, and precise intra-row weeding	[46,47,50]

A typical robotic chemical weeding system consists of four core modules: weed detection, target localization and decision-making, spray execution, and mobile platform control [40,43,49]. In the early stage of development, weed recognition relied mainly on conventional machine vision methods, such as color segmentation and morphological analysis, to identify intra-row and inter-row weeds and to trigger solenoid-valve-based spot spraying [40,43,51]. With the introduction of deep learning and edge computing, these systems increasingly adopted object detection, semantic segmentation, and lightweight convolutional neural networks, substantially improving recognition robustness under variable illumination, occlusion, and complex field backgrounds [46,49,50]. As a result, robotic chemical weeding is progressing from area-based variable-rate spraying toward plant-level precision application [46,47,49,50].

Compared with mechanical weeding, robotic chemical weeding often offers advantages in scalability, working width, and engineering integration, although these advantages should be interpreted conditionally rather than absolutely [41,43,49]. Because the system does not require direct physical contact with each target plant, it is generally more tolerant of complex crop architectures, partial occlusion, and moderate geometric positioning errors than intra-row mechanical actuators that must synchronize crop avoidance with precise tool insertion in a narrow workspace [41,46,49]. In addition, chemical weeding systems can usually be integrated more readily with existing spray booms or multi-nozzle architectures, which facilitates the expansion of working width and supports relatively high field capacity in large-scale operations [41,44,49]. From an engineering perspective, this compatibility with mature spraying hardware makes robotic chemical weeding one of the most practically deployable approaches among current robotic weed-control technologies, especially for large-area field applications [7,43,49]. However, these comparative advantages depend strongly on weed density, spray resolution, crop–weed overlap, travel speed, environmental conditions, and the agronomic context and therefore should preferably be evaluated using normalized indicators such as herbicide saving rate, field capacity, target hit rate, crop injury rate, and cost or energy input per treated area [44,46,47,49].

Despite enabling precise herbicide application, chemical weeding remains constrained by its intrinsic properties [7,41]. (1) It is subject to biological limitations. Its effectiveness depends on weed susceptibility to herbicides; if resistance develops or spray coverage is insufficient, precise localization alone cannot ensure satisfactory control [7,40,41]. (2) It is vulnerable to environmental interference. Spray drift, evaporation, and rebound deposition are strongly affected by wind speed, spray distance, and vehicle speed, which may reduce application accuracy and increase non-target risks [44,49]. (3) False detection poses a serious risk. In plant-specific spraying, misclassifying a crop as a weed can directly cause herbicide injury, making the system’s tolerance for false positives extremely low [46,49].

Accordingly, the evaluation of robotic chemical weeding systems should not be limited to algorithmic metrics such as precision or mean average precision (mAP) but should focus on a more fundamental question: whether detection results can be translated into effective field-level weed control [46,49]. High detection accuracy can only lead to high weed mortality when perception coordinates, nozzle timing, motion compensation, and spray fluid dynamics are properly matched. This application-oriented, system-level evaluation is especially critical for high-resolution microspray systems [46,47,50].

With the maturation of high-resolution microjet spraying technology, chemical weeding is shifting from simply reducing the treated area to improving target-level precision [47,49,50]. However, within the broader context of sustainable agriculture, it should not be viewed as a standalone solution but rather as a component of integrated weed management. The dominant future direction will likely be multimodal collaborative systems that combine chemical methods with mechanical, laser, electrical, or thermal non-chemical approaches to address herbicide resistance and meet the growing demand for environmentally sustainable weed control [7,43,49]. From a review perspective, robotic chemical weeding is therefore not simply a matter of mounting a sprayer on a robot; rather, it is a systems engineering problem involving the deep coupling of perception, spray fluid dynamics, motion control, and biological weed response, and further breakthroughs will require coordinated optimization across the entire pipeline [46,49,50].

### 2.3. Thermal Weeding

Thermal weeding is a key non-chemical approach in robotic site-specific weed control. Its core principle is to use high temperature or directed energy to rapidly damage weed cell structures and growing points, thereby suppressing growth [6,52,53,54,55]. Unlike chemical weeding, thermal weeding does not rely on herbicidal active ingredients, which makes it particularly attractive in organic agriculture, reduced-chemical production systems, and the management of herbicide-resistant weeds [52,54,55].

Technically, thermal weeding generally includes flame weeding, steam weeding, hot-water weeding, hot-foam weeding, and infrared weeding. Although their mechanisms differ, these methods primarily act by directly heating aboveground plant tissues, causing rapid expansion of cell fluids, membrane destabilization, and tissue necrosis, without requiring the plant to be fully burned to a carbonized state [6,17,52,53]. More specifically, flame weeding applies a brief thermal shock generated by fuel combustion to weeds at the seedling stage, with the objective of inducing rapid physiological inactivation rather than sustained burning. Steam and hot-water weeding rely on conductive heat transfer between the heated medium and the plant surface to produce thermal shock; although these systems tend to be heavier and more energy-intensive in terms of direct field energy input, they substantially reduce the risks associated with open flames. Hot-foam weeding should be distinguished from hot-water treatment because the insulating foam layer prolongs heat residence on the target surface and may improve weed control at lower water doses than hot water alone. Infrared weeding uses radiative heating elements and offers the advantages of flameless operation and controllable heat sources, but it still faces challenges in field operating speed and energy-use efficiency [6,17,52,53]. At the same time, comparisons between thermal and chemical methods should not rely only on direct field energy demand, because indirect energy embodied in herbicide production, transport, and application may also be substantial. In addition, recent developments in flame weeding include biomass-fueled or syngas-based systems as alternatives to conventional LPG, while solar-assisted pre-heating of process water may represent a potential engineering strategy for reducing the external energy demand of hot-water or hot-foam systems. Table 3 summarizes the differences among these thermal weeding routes in terms of operating mode, principal advantages, main limitations, and most suitable application scenarios, thereby providing a clear basis for technology selection. Overall, these conventional thermal methods are most effective against small seedling-stage weeds with exposed growing points. By contrast, perennial, deep-rooted, or morphologically protected weeds typically require higher energy input or repeated treatments.

In robotic systems, research on thermal weeding is shifting from broad-area thermal treatment toward site-specific thermal application tightly coupled with machine vision [6,17,19]. The essence of this transition is that the robotic platform first identifies weed locations through vision or multimodal perception, then precisely localizes the geometric center or growing point of each target and controls the heat source to act only on the weed while minimizing heat exposure to the crop [6,17]. For flame, infrared, and steam/hot-water approaches, this closed-loop process of perception, decision-making, and actuation can substantially reduce the treatment area, lower unnecessary energy consumption, and greatly improve adaptability to the confined space within crop rows [6,52].

Compared with mechanical weeding, one major advantage of thermal weeding is its non-contact mode of action [6,17,52]. Unlike blades, the heat source does not need to enter narrow intra-row spaces or withstand direct cutting loads, which reduces the risk of crop injury caused by mechanical collision during near-crop operations. In addition, thermal weeding avoids spray deposition and chemical residue, making it particularly attractive for high-value vegetables, organic crops, and production systems with strict restrictions on pesticide use [54,55]. Thermal weeding modules can also be naturally integrated with autonomous navigation, target detection, and pose-control modules, making them an important component of multimodal robotic weeding platforms [6,17].

However, thermal weeding also has several important limitations [6,17,52,53,54,55]. First, conventional flame, steam, and hot-water systems generally exhibit high energy consumption per unit area, especially when high travel speed and large working widths are required; under such conditions, energy supply and thermal efficiency become key constraints on practical deployment [53,54,55]. Existing studies indicate that, in large-scale operations, thermal weeding often requires greater energy input than chemical or mechanical alternatives [55]. Second, treatment efficacy is highly dependent on weed growth stage: seedling-stage weeds are more susceptible to thermal shock, whereas larger plants or perennial weeds may require higher doses or repeated treatment [52,54]. Third, in fields with heavy straw cover, abundant dry residues, or dry environmental conditions, flame-based thermal weeding also poses fire hazards and residue-safety concerns, which must be carefully evaluated in conservation agriculture systems [53,55].

From a systems-evaluation perspective, the performance of thermal weeding robots should not be assessed solely by whether weeds are “burned to death.” It should instead be evaluated comprehensively in terms of target hit rate, energy consumption per target, operating speed, need for repeated treatment, risk of crop injury, and environmental safety [6,17,55,58]. For flame, steam, and hot-water systems, there is typically a clear trade-off between heat-transfer efficiency and operating speed: as travel speed increases, the exposure time per target decreases, which may reduce control efficacy; conversely, lowering speed to increase thermal dose may compromise overall field efficiency and energy-use efficiency [53,54,55]. Thermal weeding should therefore be understood not simply as a matter of heat-source delivery but as an integrated systems-engineering problem involving target recognition, thermal energy transfer, motion control, and biological response [6,17,58].

Within the framework of sustainable agriculture and integrated weed management, the practical value of thermal weeding lies primarily in combining its non-chemical nature with the precision-control potential of robotic systems [6,17,53]. For production systems that cannot tolerate chemical residues, thermal weeding offers a relatively direct physical control pathway [53,54]. Over the medium to long term, however, thermal weeding is more likely to serve as a key component of multimodal precision weeding systems, used in combination with mechanical, chemical, or electrical approaches rather than fully replacing all other interventions [6,17]. Overall, thermal weeding combines strong potential for environmentally sustainable weed control with substantial engineering challenges. Future development will likely focus on improving energy-use efficiency, enhancing target-level precision, reducing system cost, and strengthening safety protection mechanisms [6,17,19,53,58,59,60].

### 2.4. Laser-Based Weeding Modalities

Laser weeding, as a frontier technology in robotic precision weed control, is driving a paradigm shift from selective treatment to plant-level targeted intervention [19,58,61,62,63]. Its core principle is to use a high-energy-density laser beam to inflict localized thermal damage on the weed growing point or other critical tissues within a very short time, thereby disrupting cellular structure and suppressing growth [19,58,63]. Owing to its dual advantages of non-contact operation (avoiding mechanical crop injury) and non-chemical action (eliminating spray residues), laser weeding has shown particular promise in organic agriculture and the management of herbicide-resistant weeds, especially for near-crop operations in high-value vegetable and orchard systems [19,59,61,62].

A typical laser weeding system is a complex closed-loop platform integrating perception, decision-making, control, and actuation [19,58,59,60,64,65]. The system first uses deep learning algorithms to accurately detect weeds and locate their growing points in complex field backgrounds and then maps the visual coordinates in real time to the laser-emission coordinate system. A key requirement is robust dynamic compensation to offset delays caused by robot motion, image processing, and mechanical response so that the laser beam can maintain stable target engagement even during high-speed operation [58,59,65]. Accordingly, the performance of laser weeding depends not only on the accuracy of the recognition algorithm but also on the combined effectiveness of coordinate calibration, gimbal response speed, and motion-control strategy [19,58,59]. Table 4 provides a detailed comparison of different systems in terms of target site, system configuration, and their respective advantages and limitations.

Despite its clear advantages, the engineering deployment of laser weeding still faces substantial challenges [19,58,59,60,64,65,70]. First, it imposes exceptionally stringent accuracy requirements: any coordinate deviation caused by occlusion or illumination changes may result in missed targets or unintended crop injury [19,58,59,64]. Second, high equipment cost, complex thermal and power management, and relatively low operational throughput limit its applicability in large-scale production of low-value crops [58,59,65]. Most critically, safety remains a major concern. Potential eye injury, reflected-beam hazards, and fire risk require laser weeding systems to incorporate multilayer safety architectures, including enclosed workspaces, target interlocks, emergency-stop mechanisms, and intrusion detection [19,60,63,70].

From an agronomic perspective, the effectiveness of laser weeding is highly sensitive to treatment timing. It performs best at the seedling stage, particularly from the cotyledon to two-leaf stage, when the growing point is exposed and the required energy input is relatively low; by contrast, for larger weeds, both control efficacy and energy efficiency decline markedly [61,62]. This indicates that laser weeding has clear agronomic timing requirements and is better suited as an early precision-intervention tool rather than as a complete replacement for later-stage control of larger weeds [61,62,63].

In terms of research progress, laser weeding has evolved from early static prototype validation to mobile robotic systems that integrate deep-learning-based detection, dynamic path planning, and autonomous navigation [19,58,59,60,64,65,70]. Although it is unlikely to replace all conventional weeding methods, it holds an irreplaceable role in high-value, low-density, and zero-residue production scenarios. The most likely future direction is its integration into multimodal collaborative weeding systems, where it complements mechanical, chemical, and other control methods to address complex field conditions [19,62,63,70]. Overall, laser weeding is not merely a novel end-effector but a systems-engineering challenge requiring the deep integration of artificial intelligence, optoelectromechanical systems, motion control, and weed biology [19,58,63,70].

### 2.5. Electrical Weed Control Modalities

Electrical weeding is a highly promising non-chemical approach in robotic weed management. Its core principle is to deliver high-voltage or otherwise controlled electrical energy into the plant through electrode contact, thereby destroying tissues and causing plant death through mechanisms such as Joule heating and cellular rupture [6,73,74,75,76,77]. Owing to its lack of chemical residues, minimal soil disturbance, and potential to damage the root systems of perennial weeds, electrical weeding has attracted increasing attention in organic agriculture and in the management of herbicide-resistant weeds [6,73,78]. The dominant current technical route is continuous electrode-contact treatment. Rather than a simple process in which “electricity kills on contact,” it is better understood as an energy-transfer process jointly governed by multiple factors, including power level, operating speed, plant water content, and soil electrical conductivity [73]. Table 5 summarizes the differences among major electrical weeding approaches in terms of operating mechanism, advantages, limitations, and application scenarios [73,74,78,79].

In robotic systems, electrical weeding is shifting from broad-area treatment toward vision-guided, site-specific precision contact [6,79]. In these systems, the perception module identifies weeds, and the control system guides the electrode to make precise contact with the target before delivering electrical energy. This process depends heavily on front-end detection accuracy, electrode pose control, and motion compensation; any poor contact or positional deviation may reduce treatment efficacy or cause unintended crop injury. Particularly important is the strong sensitivity of electrical weeding to both species and growth stage: dicot weeds are generally more susceptible, and the energy required at the seedling stage is far lower than that required for mature plants [73,74,81]. This indicates that future robotic systems must support dynamic parameter optimization, adjusting energy output in real time according to target type rather than relying on fixed power settings.

Despite its considerable potential, the practical deployment of electrical weeding still faces major engineering challenges [73,78,79]. First, its effectiveness is strongly influenced by environmental conditions: water films on plant surfaces may cause current bypass, while variation in soil conductivity affects energy distribution [73,78]. Second, safety is a central constraint. High-voltage output requires rigorous insulation, interlock, and emergency-stop mechanisms, and it may also create fire risks in dry, residue-covered environments [73,78]. Third, system integration remains complex, as high-power delivery must be balanced against the need for lightweight robotic platforms [73,79].

Overall, electrical weeding is a highly promising and forward-looking non-chemical technology in robotic weed management. Its main advantages lie in the absence of chemical residues, its strong compatibility with precision perception, and its particular potential against some perennial or regenerative weeds [6,73,78,82]. However, its ultimate performance remains constrained by multiple interacting factors, including species differences, growth stage, contact quality, energy efficiency, environmental conditions, and the stringent safety requirements associated with high-voltage operation [73,78,81]. Electrical weeding should therefore be regarded not as a universal replacement for all other methods but as a key complementary technology within integrated weed management, working in multimodal synergy with mechanical, laser, and precision spraying approaches [6,78,79].

### 2.6. Other Emerging Weeding Approaches

Beyond the mainstream approaches of mechanical, chemical, thermal, laser, and electrical weeding, several emerging alternatives, including microwave weeding, bioherbicides, and cold plasma treatment, are beginning to attract attention and offer new possibilities for sustainable weed management [83,84,85,86,87]. Although these methods are generally less mature than the major weeding modalities discussed above and remain largely at the stage of experimental validation or prototype development, they collectively point to an important trend: reducing dependence on chemical control while building a more diversified and environmentally sustainable weed management system through deep integration with precision detection, site-specific application, and intelligent robotic platforms [83,84,85,87]. Table 6 briefly summarizes the mechanisms of action, core advantages, main limitations, and current development status of these emerging approaches.

Microwave weeding exploits dielectric heating to rapidly raise the temperature of water within plant tissues and soil, thereby killing weeds [83,84]. Unlike conventional surface heating, microwave treatment provides volumetric heating, enabling it not only to destroy aboveground tissues but also potentially to affect the shallow soil seed bank and underground propagules [83]. However, its application in large-scale conventional field settings remains limited by high energy demand, difficulty in controlling treatment depth, and the pronounced trade-off between power density and operating speed. At present, it is therefore more suitable for localized intensive treatment in high-value production systems [84].

Bioherbicides represent a different non-physical approach, relying on microorganisms or natural metabolites to selectively suppress weeds [85]. Although their environmental compatibility and target specificity are widely recognized, their broader adoption has long been constrained by unstable activity, poor environmental adaptability, slow onset of action, and difficulties in formulation and commercialization [85,86]. From the perspective of robotic application, a promising route forward is precision site-specific delivery: vision systems could apply microdoses only to identified weed individuals or hotspot areas, thereby substantially improving utilization efficiency while minimizing non-target risk. This may become a key pathway for the engineering translation of bioherbicides [86].

Cold plasma is another emerging approach. It damages seeds or seedling tissues through reactive oxygen and nitrogen species together with localized electrothermal effects and in theory offers a residue-free control strategy [87]. Existing studies suggest that cold plasma can be used both to inhibit seed germination and to treat small emerged weeds, indicating considerable potential as a residue-free technology [87]. However, this approach remains at a relatively early stage of development. Sufficient evidence is still lacking regarding field-scale implementation, device stability, and energy efficiency, and it is still far from becoming a mature robotic site-specific weeding solution. It is therefore more appropriate to include it in a review as an emerging frontier rather than to discuss it alongside established technologies [87].

Overall, although these emerging technologies share common challenges, including high energy consumption, limited stability, and a lack of standardization, they substantially expand the weed-management toolbox and reflect a broader future trend: a shift from reliance on a single type of end-effector toward multimodal physical and biological intervention strategies [83,84,85,86,87].

## 3. Sensing Requirements Across Weeding Modalities

**Figure 4:**
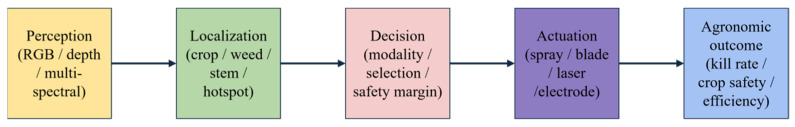
General sensing–actuation coupling framework for robotic weed management.

Robotic weeding systems operate through a tightly coupled pipeline that links environmental perception to physical intervention [69,88]. While significant research effort has been devoted to improving weed detection algorithms, the sensing requirements of robotic weeding systems are fundamentally determined by the characteristics of the intervention modality [89]. Figure 4 presents the general sensing–decision–actuation pipeline in robotic weeding systems and shows how perception outputs are translated into physical weed control actions. Different weeding approaches impose different demands on the spatial precision, output format, and robustness of perception results [90]. For example, some modalities require only coarse localization of weed presence within a treatment zone, whereas others demand millimeter-level positioning accuracy or even estimation of specific plant structures such as the stem or growth point [67].

### 3.1. Chemical Spot Spraying

Chemical spot spraying represents one of the most widely deployed forms of site-specific weed management [46,48,91]. Compared with other robotic weeding modalities, this approach generally imposes relatively moderate requirements on perception precision [46,48,92]. In many systems, weed localization through bounding boxes or centroid coordinates is sufficient to guide herbicide application [46,91]. The spraying mechanism typically covers a small treatment area rather than targeting a single precise impact point, which provides tolerance to moderate localization errors [48,91,93].

**Figure 5:**
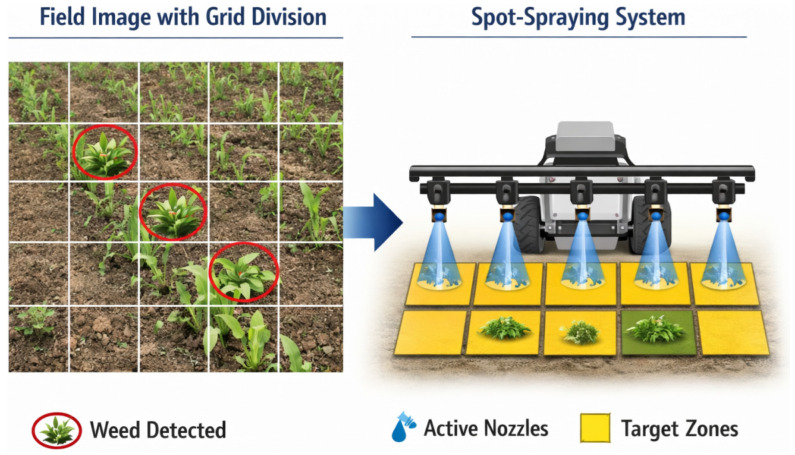
Grid-based perception–actuation mapping strategy for robotic spot spraying.

In practical implementations, perception outputs are often converted into spray control zones rather than individual plant coordinates [50]. One implementation strategy is to divide the captured field image into a grid structure, where each grid cell corresponds to the physical coverage area of a spray nozzle [94]. Figure 5 illustrates a grid-based perception–actuation mapping strategy, in which weed detections are converted into nozzle-specific spray control zones. When weeds are detected within a particular grid cell, the corresponding nozzle is activated as the robot passes over the target area [95]. This grid-based mapping approach simplifies the perception–actuation interface and allows efficient real-time control even when detection results are relatively coarse [95,96]. As a result, spot spraying systems can operate effectively with object detection or classification-based perception outputs without requiring detailed plant morphology information [97].

### 3.2. Laser Weeding Sensing Requirements

Laser-based weeding systems impose substantially stricter requirements on perception accuracy [69,98]. Unlike spraying systems that treat a small area, laser ablation typically concentrates energy on a very small target point on the weed plant [69,98]. As a result, even minor localization errors may lead to ineffective weed damage or unintended crop injury [99,100]. Therefore, many laser weeding platforms require high-precision estimation of weed positions, often with millimeter-level spatial accuracy [67,101].

In addition to basic plant detection, some systems attempt to estimate specific plant structures such as the stem base or the apical growth point, which represent the most effective targets for laser ablation [66,68]. Identifying these biologically sensitive locations improves the lethality of the laser treatment while minimizing energy consumption [89]. Consequently, perception pipelines for laser weeding frequently rely on high-resolution segmentation models, precise geometric calibration between the camera and laser actuator, and real-time coordinate transformation to ensure accurate targeting during robot motion [69,71,72].

### 3.3. Mechanical Intra-Row Weeding

Mechanical intra-row weeding systems often place stronger emphasis on accurate crop localization rather than direct weed identification [36]. Since many mechanical tools physically disturb the soil near crop plants, avoiding crop damage becomes the primary constraint [36]. As a result, perception systems in these platforms commonly focus on detecting crop centers, estimating stem positions, or reconstructing the spatial boundary between crop plants and surrounding weeds [102].

In some implementations, mechanical actuators such as knives, rotating hoes, or stamping tools operate within narrow zones between crop stems [36]. Therefore, perception outputs must provide reliable estimates of crop positions with sufficient temporal stability to support synchronized actuation during robot movement [102]. Techniques such as crop row detection, plant center localization, and stem detection are frequently employed to maintain safe operating margins around the crop plants while enabling effective removal of weeds within the remaining soil area [67].

### 3.4. Thermal Broadcast Treatment

Thermal weed control methods, including flame weeding, hot water treatment, and infrared heating, typically operate in a broadcast or semi-broadcast manner [103]. In many cases, these approaches apply heat over a continuous treatment band rather than targeting individual plants [52]. Consequently, the perception requirements for such modalities are generally less stringent compared with highly targeted methods such as laser ablation [104].

For example, thermal treatments used during pre-emergence weed control may not require individual weed detection at all [105]. Instead, perception systems may focus on identifying crop rows or distinguishing treated zones from crop areas to prevent unintended crop exposure [57]. In selective thermal systems, coarse localization of weed clusters or treatment strips may be sufficient to guide actuator activation [106]. As a result, perception outputs for thermal weeding often emphasize robustness and coverage rather than extremely high spatial precision [103].

### 3.5. Electrical Weeding Sensing Requirements

Electrical weed control systems deliver high-voltage electrical energy through electrodes that contact or approach the weed plant [107]. The effectiveness of this modality depends on establishing sufficient electrical conduction through the plant tissues, which often requires physical contact with the weed stem or foliage [108]. Consequently, perception systems for electrical weeding typically require reliable detection of plant locations and approximate plant height or structure to ensure effective electrode positioning [82].

Compared with laser systems, electrical weeding generally tolerates slightly lower spatial precision because the electrode often interacts with a larger portion of the plant structure [80]. However, perception must still distinguish weeds from crops with high reliability to avoid unintended damage [109]. Therefore, perception pipelines for electrical weeding frequently combine plant detection with geometric filtering or crop-row constraints to ensure safe actuator deployment [107].

## 4. Cross-Modality Comparison and Scenario-Based Decision Framework

**Figure 6:**
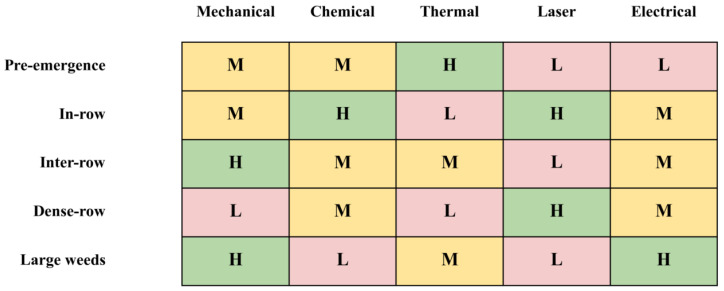
Scenario–modality suitability matrix for robotic weed management. The matrix qualitatively compares the relative suitability of major weeding modalities across representative agricultural scenarios, where H, M, and L represent high, moderate, and low relative suitability, respectively. The comparison is derived from the reviewed literature and is intended to support scenario-oriented system design rather than provide a quantitative or absolute performance ranking.

While the previous sections describe the technical characteristics of individual weeding modalities and their associated sensing requirements, the practical deployment of robotic weeding systems is ultimately determined by field conditions and agronomic scenarios [110]. Different cropping systems, weed growth stages, and operational constraints require different intervention strategies [111]. Therefore, selecting an appropriate weeding modality is not merely a technical decision but a context-dependent process that must balance effectiveness, crop safety, operational efficiency, and energy consumption [112]. This section analyzes the suitability of different weeding modalities across several representative agricultural scenarios and provides a scenario-oriented comparison framework to guide system design and technology selection [113]. Figure 6 summarizes the relative suitability of different weeding modalities across representative agricultural scenarios and provides a scenario-oriented comparison framework for modality selection.

### 4.1. Pre-Emergence Weed Control

Pre-emergence weed control targets weeds before or shortly after germination, when weed seedlings are small and crop plants have not yet emerged or are still highly tolerant to disturbance [114]. Under such conditions, broad-area treatments are often sufficient because the risk of crop damage is relatively low [115]. Thermal approaches such as flame weeding, hot water treatment, steam application, and infrared heating are widely considered suitable for this stage, as they can effectively destroy young weed tissues through rapid temperature elevation [56]. These methods are particularly attractive because they can reduce herbicide use while covering relatively large treatment areas [114].

Compared with highly targeted robotic approaches, pre-emergence weed control generally requires less precise sensing and actuation [114]. In many cases, perception systems only need to identify crop rows or determine treatment zones to avoid damaging newly emerging crops [115]. As a result, broadcast or band-based thermal treatments can achieve high operational efficiency, making them suitable for early-season weed suppression in large-scale cropping systems [114].

### 4.2. In-Row Selective Weeding

In-row selective weeding represents one of the most technically challenging scenarios in robotic weed management, as weeds must be removed in close proximity to crop plants [116]. In such situations, the primary challenge lies in achieving high removal efficiency while avoiding crop injury [36]. Precision-targeted modalities such as laser weeding, spot spraying, and certain mechanical intra-row tools are commonly explored for this purpose [27]. Laser-based systems, in particular, offer the ability to deliver highly localized energy to individual weed plants, enabling selective control even within densely planted crop rows [71].

However, the success of in-row weeding strongly depends on the accuracy of plant localization and the response speed of the actuation mechanism [117]. High-resolution perception systems are often required to estimate crop stem positions or plant centers in real time [25]. Mechanical intra-row tools may rely on precise crop localization to maintain safe distances around crop stems, while laser systems may require millimeter-level targeting accuracy [118]. Consequently, in-row weeding systems often represent the most demanding integration of perception, planning, and actuation within robotic weed management [113].

### 4.3. Inter-Row Weeding

Inter-row weeding targets weeds growing between crop rows, where the spatial separation between crops and weeds is larger and the risk of crop damage is relatively lower [119]. This scenario allows the use of more aggressive mechanical tools and wider treatment zones compared with in-row operations [119]. Mechanical cultivation implements such as rotating hoes, brush weeders, and inter-row knives are widely used because they can disturb the soil and uproot weeds effectively while maintaining safe distances from crop rows [119].

Because crop rows provide strong geometric cues, perception requirements in inter-row weeding are generally less demanding than those in in-row scenarios [112]. Many robotic systems primarily rely on crop-row detection or row-following algorithms to guide mechanical actuators between rows [112]. This approach allows relatively high operating speeds and makes inter-row mechanical weeding one of the most mature and practically deployable robotic weed control strategies in current agricultural robotics systems [119].

### 4.4. Dense-Row Crop Systems

Dense-row crop systems, such as carrots, onions, and certain leafy vegetables, present unique challenges for robotic weed control due to the small spacing between crop plants [112]. In these systems, the limited available workspace significantly restricts the types of intervention mechanisms that can be applied [112]. Mechanical tools that require large operating clearance may become unsuitable, and crop–weed discrimination becomes more difficult due to plant overlap and similar morphology [119].

Under such conditions, highly localized interventions such as tube stamping mechanisms, microdosing chemical spraying, and laser-based weeding have been explored [27]. These modalities can operate within narrow spatial margins while minimizing disturbance to surrounding crops [27]. However, dense-row systems often demand higher perception accuracy and faster actuator response times, making them a particularly challenging but important application domain for advanced robotic weeding technologies [112].

### 4.5. Large and Perennial Weeding Situations

Large weeds and perennial species with well-developed root systems present additional challenges for robotic weed management [120]. Unlike small seedlings, mature weeds often require stronger mechanical or electrical interventions to achieve effective control [75]. Methods such as electrical weeding, mechanical uprooting, or cutting tools can provide sufficient energy or force to damage both above-ground and root structures [120].

However, targeting larger weeds also introduces additional operational constraints [79]. For example, electrical weeding systems must ensure proper contact between electrodes and plant tissues to achieve effective current flow, while mechanical removal tools must generate sufficient force to uproot deeply rooted plants [75]. In such cases, perception systems must not only detect weed locations but may also need to estimate plant size or structural characteristics to select appropriate intervention strategies. As a result, large weed control often favors robust, high-energy modalities capable of handling heterogeneous plant structures [75].

### 4.6. Quantitative Performance Indicators Reported for Robotic Weeding Modalities

Although the scenario-based comparison above helps clarify the relative suitability of different weeding modalities, the practical value of a robotic weeding system ultimately depends on measurable engineering and agronomic indicators. These indicators include operating speed, field capacity, energy or chemical input, positioning accuracy, weed control efficacy, crop injury rate, labor reduction, and treatment cost. However, quantitative comparison across studies remains challenging because reported values are strongly affected by crop type, weed species, weed density, weed growth stage, soil condition, actuator configuration, platform speed, and evaluation protocol. Therefore, the values summarized in Table 7 should be interpreted as representative performance ranges or case-study indicators rather than absolute rankings among modalities.

Table 7 shows that mechanical intra-row weeding systems can achieve high weed removal efficacy under favorable crop-spacing and soil conditions, but their performance is strongly constrained by travel speed and crop avoidance accuracy. For example, robotic intra-row cultivators have been reported to remove 18–41% more weeds and reduce subsequent hand-weeding time by 20–45% compared with standard cultivation under moderate to high weed densities [121]. More recent intelligent intra-row devices have reported weeding accuracies above 95% at low operating speeds, but crop injury increased and weeding accuracy declined as travel speed increased [36]. These results confirm that mechanical weeding performance is governed by a speed–precision trade-off.

Chemical spot spraying generally shows stronger scalability and higher field applicability when the primary goal is to reduce herbicide input without compromising weed control efficacy. In sugarcane field trials, a robotic spot-spraying system achieved weed control equivalent to 97% of broadcast spraying while reducing herbicide use by 35% on average and by up to 65% in low-weed-pressure strips [48]. This indicates that the main quantitative advantage of chemical site-specific weeding lies in chemical input reduction rather than complete elimination of herbicide use.

For thermal methods, quantitative performance is mainly reflected in fuel, water, or heat dose requirements. Hot-foam treatment studies have shown that doses of 3.33–8.33 L m^2^ can achieve complete weed cover devitalization under specific weed compositions, whereas higher doses such as 6.67–8.33 L m^2^ may be required to delay regrowth in more difficult weed communities [103]. Compared with hot water alone, adding foam can reduce the required water dose by at least 2.5-fold because the insulating foam layer increases peak temperature and slows heat dissipation [122]. These results support the view that hot foam is not simply equivalent to hot water, but represents a distinct thermal-delivery strategy with improved heat residence on the target surface.

Laser weeding provides the highest spatial selectivity among the major physical weeding modalities, but its quantitative performance depends heavily on target size, growth stage, beam diameter, and delivered energy. In a recent dose–response study using a 50 W thulium-doped fiber laser with a 2 mm beam diameter and 2 µm wavelength, weeds were exposed to doses from 0.4 to 12.7 J mm^2^, corresponding approximately to 1.3–39.9 J per 2 mm laser spot [61]. The highest efficacy was achieved when grass weeds were at the one-leaf stage and dicotyledonous weeds were at the cotyledon stage, whereas many species showed regrowth at the four-leaf stage [61]. This demonstrates that laser weeding should be regarded as an early-stage precision intervention rather than a universal high-throughput solution for mature weeds.

Electrical weeding shows strong potential for non-chemical control, particularly when energy can be delivered efficiently to young weeds. In greenhouse trials using pulsed microshocks, 100% control of several broadleaf weed seedlings was achieved with estimated energy requirements of 0.1–0.9 MJ ha^−1^ for a weed density of five plants m^2^, whereas grass weeds were less susceptible [80]. Subsequent outdoor field experiments with a flat-plate electrode achieved better than 90% control for all tested species, with energy requirements ranging from 15 kJ ha^−1^ for some broadleaf species to 363–555 kJ ha^−1^ for in-ground Lolium multiflorum depending on electrode placement [123]. These values are considerably lower than those of many broad-area thermal methods, but the method remains sensitive to species, growth stage, contact quality, and soil moisture.

Overall, the quantitative indicators reported in Table 7 further reinforce the central argument of this review: no single modality can be evaluated using one universal performance metric. Mechanical weeding should be assessed by weed removal rate, crop injury, and operating speed; chemical spot spraying by herbicide saving rate and target hit rate; thermal methods by heat dose and regrowth delay; laser weeding by energy per target and targeting accuracy; and electrical weeding by delivered energy, contact reliability, and plant mortality. Future studies should therefore report standardized indicators, including weed mortality, crop injury rate, operating speed, energy or chemical input per treated area, positioning error, treatment cost, and repeated-treatment requirement, so that robotic weeding systems can be compared more rigorously across platforms and agricultural scenarios.

## 5. Multi-Modality Integration and Future Directions

### 5.1. Limitations of Single-Modality Systems

Although significant progress has been achieved in the development of robotic weeding technologies, most current systems rely on a single intervention modality [48]. In practice, however, each modality exhibits inherent limitations when applied across diverse agricultural scenarios [59]. Mechanical weeding can effectively remove weeds through soil disturbance or uprooting, but it may cause crop damage in dense planting systems or when crop–weed spacing is limited [124]. Chemical spot spraying enables targeted herbicide application and reduces chemical usage compared with broadcast spraying, yet concerns regarding herbicide resistance, environmental impact, and regulatory restrictions continue to limit its long-term sustainability [48].

Similarly, laser-based weeding systems provide highly precise and chemical-free weed removal, but they often face challenges related to energy consumption, operational speed, and limited effectiveness against larger or perennial weeds [61]. Thermal and electrical approaches may deliver sufficient destructive energy to weeds but can be constrained by energy efficiency, safety considerations, or limited selectivity in certain field conditions [63]. As a result, no single weeding modality can fully satisfy the diverse requirements of modern crop production systems, particularly under heterogeneous field conditions and varying weed growth stages [63].

### 5.2. Hybrid Weeding Strategies

**Figure 7:**
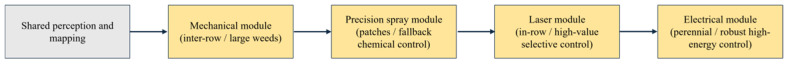
Conceptual architecture of a hybrid robotic weeding platform integrating multiple intervention modalities. Different actuators can be selectively activated according to local crop–weed configuration, weed growth stage, and field accessibility, thereby improving operational flexibility and overall control effectiveness.

Given the limitations of individual intervention approaches, increasing research attention has been directed toward hybrid weeding strategies that integrate multiple modalities within a single robotic platform [90]. By combining complementary mechanisms, hybrid systems aim to improve overall weed control effectiveness while maintaining operational flexibility [90]. To address the limitations of single-modality systems, Figure 7 presents a conceptual hybrid robotic weeding architecture in which complementary intervention mechanisms are coordinated within one platform. For example, integrating precision spraying with mechanical tools allows robots to apply herbicides only where mechanical removal is infeasible, while mechanical cultivation can reduce herbicide dependence in inter-row areas [125].

Similarly, laser-based systems can be combined with chemical or mechanical approaches to address weeds at different growth stages or spatial positions [90]. In such configurations, highly targeted laser treatment may be used for in-row weed removal, while mechanical or spraying mechanisms handle inter-row or dense weed patches [90]. These hybrid architectures allow robotic platforms to dynamically select the most appropriate intervention modality based on local field conditions, thereby improving system robustness and expanding applicability across diverse cropping environments [6].

### 5.3. Air-Ground Collaborative Weed Management

**Figure 8:**
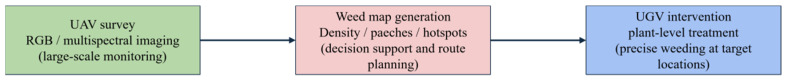
Conceptual air–ground collaborative framework for robotic weed management. UAVs perform large-scale field scouting and weed mapping, while ground robots use aerially derived information to conduct targeted physical interventions at the local scale.

Beyond integrating multiple intervention mechanisms, future robotic weed management systems may increasingly adopt air–ground collaborative architectures [126,127]. Figure 8 shows a conceptual air–ground collaborative weed management framework in which aerial platforms provide large-scale field monitoring and decision support, while ground robots perform precise plant-level interventions. Unmanned aerial vehicles (UAVs) can rapidly survey large agricultural fields and generate high-resolution weed distribution maps using multispectral or RGB imaging combined with machine learning-based weed detection [128,129,130]. These aerial observations provide valuable information on weed density, spatial distribution, and growth patterns at the field scale [128,129].

Ground-based weeding robots can then use these maps to perform targeted interventions at the plant level, significantly improving operational efficiency compared with purely reactive perception systems [126,127,131]. Such air–ground collaboration enables a hierarchical weed management framework in which aerial platforms perform large-scale monitoring and decision support, while ground robots execute precise physical interventions [126,131]. This cooperative strategy has the potential to greatly enhance scalability and efficiency in large agricultural operations, particularly in extensive cropping systems where rapid field coverage is essential [127,131].

## 6. Discussion

Robotic weeding has experienced rapid technological progress over the past decade, driven by advances in computer vision, autonomous navigation, and precision actuation technologies. However, despite the increasing number of experimental prototypes and pilot deployments, several technological bottlenecks still limit large-scale adoption in commercial agriculture. One major challenge lies in the robustness of perception systems under highly variable field conditions. Weed detection algorithms that perform well under controlled experimental settings may suffer from reduced reliability when exposed to complex backgrounds, illumination variations, plant occlusion, and heterogeneous crop growth stages. In addition, the real-time coordination between perception, motion planning, and actuation remains technically demanding, particularly when robots operate at practical field speeds.

Another important issue concerns the trade-offs between different weeding modalities. Each intervention mechanism presents a distinct balance between precision, energy consumption, operational speed, and crop safety. Mechanical weeding can provide strong physical removal of weeds and may be effective against larger plants, but it may also increase the risk of crop disturbance in dense planting systems. Chemical spot spraying enables efficient and scalable weed control but still relies on herbicides, which raises concerns regarding environmental impact and resistance development. Laser weeding offers highly targeted and chemical-free control but is currently constrained by energy efficiency and operational throughput. Thermal and electrical methods can deliver substantial destructive energy but may face limitations in selectivity or energy requirements. These trade-offs indicate that the suitability of each modality is highly context-dependent.

The quantitative indicators summarized in Section 4.6 further indicate that modality comparison should be based on multiple normalized metrics rather than a single performance index. In particular, weed mortality, crop injury, operating speed, energy or chemical input per unit area, positioning error, and treatment cost should be reported simultaneously to support rigorous cross-platform comparison.

Beyond modality-level considerations, the current research landscape also reveals several important gaps in robotic weeding studies. Many proposed systems are evaluated only under small-scale experimental plots or controlled environments, with limited long-term field validation across diverse crop types and environmental conditions. Furthermore, there is a lack of standardized benchmark datasets and evaluation protocols for comparing robotic weed control systems across different platforms and modalities. Economic feasibility also remains insufficiently studied, as relatively few works analyze operational costs, maintenance requirements, and potential return on investment under real farming conditions.

Another challenge lies in system integration at the farm scale. Robotic weed management cannot be considered an isolated technology but should instead be integrated into broader digital agriculture ecosystems. Future systems will likely need to interact with crop monitoring platforms, farm management information systems, and autonomous logistics infrastructure. In this context, the coordination between aerial sensing platforms, ground-based robots, and cloud-based decision support systems may play an increasingly important role in enabling scalable and adaptive weed management strategies.

Overall, the development of robotic weeding technologies reflects a broader transition toward data-driven and automation-enabled farming systems. While significant technical progress has been achieved in sensing, actuation, and robotic platforms, achieving reliable, economically viable, and scalable weed management solutions will require continued interdisciplinary collaboration across agricultural science, robotics, artificial intelligence, and farm management. Addressing these challenges will be critical for transforming robotic weed control from experimental prototypes into widely adopted tools for sustainable crop production.

## 7. Conclusions

This review provides an actuation-oriented perspective on robotic weeding within site-specific weed management, shifting the focus from detection-centric approaches to the coupling between sensing and physical intervention. A unified taxonomy of weeding modalities, including mechanical, chemical, thermal, laser-based, electrical, and emerging approaches, is established, and their mechanisms and operational characteristics are systematically analyzed. By examining sensing–actuation coupling, this work clarifies how different modalities impose distinct requirements on perception outputs, ranging from coarse localization to high-precision targeting and structural estimation, highlighting that detection accuracy alone is insufficient for effective weed control. A scenario-based framework further demonstrates that no single modality can meet the diverse demands of real-world agricultural systems due to inherent trade-offs in precision, efficiency, and crop safety. By incorporating representative quantitative indicators such as operating speed, energy or chemical input, targeting accuracy, weed control efficacy, and crop injury risk, this review further links modality-level mechanisms with measurable engineering performance. These findings emphasize the need for context-aware system design and support the growing trend toward multi-modality integration and air–ground collaborative strategies. Overall, this review provides a decision-oriented reference for the design and deployment of next-generation robotic weeding systems, contributing to the advancement of sustainable and data-driven agriculture.

## Figures and Tables

**Table 1:** Representative end-effectors used in robotic mechanical weeding: typical modes of action, advantages, limitations, application scenarios, and representative references.

End-Effector Type	Typical Mode of Action	Main Advantages	Main Limitations	Typical Application Scenarios	Representative References
Fixed blade/hoe-shovel type	Cutting, shallow soil disturbance, soil covering	Simple structure, low cost, strong capacity for continuous operation	Limited precision for intra-row crop avoidance; performance is sensitive to soil hardness	Inter-row weeding; regular ridge cultivation	[23,24,27]
Oscillating/opening–closing blade type	Rapid blade insertion and retraction within gaps between crops	Strong intra-row operating capability; suitable for transplanted crops or crops with regular plant spacing	Requires high crop localization accuracy and precise timing control	Intra-row weeding in vegetables and row-planted crops	[24,25,27]
Rotary blade head/disk type	Rotary cutting, root disturbance, shallow tillage	High efficiency in controlling small to medium-sized weeds	May cause soil throwing; requires a high safety margin near crop seedlings	Inter-row and near-crop zones	[23,26,27]
Finger-weeder/spring-tooth/flexible tine type	Flicking, soil loosening, uprooting young weeds	Well suited to shallow-rooted weeds at the seedling stage; crop injury is relatively controllable	Limited effectiveness against larger weeds and deep-rooted species	Seedling-stage crops; fields with light weed infestation	[23,27]
Offset laterally shifting near-crop mechanism	Cutting close to tree trunks or crop bases	Suitable for near-trunk weeding in orchards and vineyards	Structurally and computationally complex; typically requires obstacle avoidance or trunk detection	Orchards, vineyards, and intra-row woody crops	[26]

**Table 3:** Comparison of different thermal weeding routes in terms of operating mechanism, advantages, limitations, application scenarios, and representative references.

Technical Route	Operating Mechanism	Main Advantages	Main Limitations	Typical Application Scenarios	Representative References
Flame weeding	Uses brief high-temperature exposure from an open flame to damage weed tissues and growing points	Direct action; relatively simple equipment principle; suitable for rapid treatment of seedling-stage weeds	High energy consumption; fire hazard; poorly suited to fields with dry residues or heavy straw cover	Organic agriculture, early-stage weed control, and fields with limited surface residues	[52,53,55,56]
Steam weeding	Uses heat transfer from high-temperature steam to inactivate weed tissues	Flameless operation; relatively high safety; applicable to some near-crop situations	Lower thermal efficiency and greater complexity of the heating system; equipment tends to be heavy	Protected cultivation, near-crop zones, and environments sensitive to open flames	[52,53,54,55]
Hot-water weeding	Applies high-temperature water directly to the weed surface to induce thermal shock	Uniform treatment; lower fire risk	High water demand and heating requirements; limited operational efficiency	Small-scale precision management, urban settings, or protected cultivation	[52,54,57]
Infrared weeding	Transfers heat to weeds through infrared radiation elements	Controllable heat source; no direct flame; theoretically easy to modularize	Limited by operating speed and energy-use efficiency; field adaptability is affected by environmental conditions	Small-scale field operations and targeted thermal treatment	[52,53]
Hot-foam weeding	Applies hot water with an insulating foam layer to prolong heat residence on weed tissues	Higher heat-retention efficiency than hot water alone; may reduce water dose and improve treatment uniformity	Requires heated water, foaming agent, and additional equipment; still energy-intensive and generally non-selective	Organic or residue-sensitive systems, protected cultivation, urban/non-crop areas, and localized high-value crop applications	[52,53,55]

**Table 4:** Comparison of the main technical characteristics, advantages, limitations, application scenarios, and representative references of laser weeding systems.

Comparison Dimension	Main Content	Main Advantages	Main Limitations	Typical Application Scenarios	Representative References
Mechanism of action	Uses a high-energy-density laser beam to inflict localized thermal damage on the weed growing point, stem base, or other critical tissues	Small treatment zone, high spatial selectivity, and plant-level control	Requires extremely high targeting accuracy; improper energy settings may lead to incomplete kill or regrowth	Seedling-stage weeds and fine-scale individual plant treatment	[19,55,58,61,62,63]
Perception and recognition	Identifies crops, weeds, and growing points using machine vision, deep learning, or keypoint detection	Easily integrated with AI-based recognition modules and well suited to precision operations	Recognition stability decreases under occlusion, variable illumination, or complex backgrounds	Vegetables, orchards, and intra-row weeding in regularly planted systems	[58,66,67,68,69]
Coordinate transformation and execution	Maps image coordinates to laser-emission coordinates and triggers the laser with speed compensation	Enables true post-detection targeted intervention	Calibration errors, platform vibration, and system latency directly affect hit accuracy	Mobile robots and autonomous navigation platforms	[58,65,70,71,72]
Operational characteristics	Non-contact, chemical-free, and without soil disturbance	No chemical residues, no soil disruption, and well suited to reduced-chemical and organic systems	Limited throughput; efficiency declines when weed density is high	High-value crops and fields with low to moderate weed pressure	[59,61,62,63]
Engineering implementation	Requires coordinated integration of the laser source, thermal management, power supply, safety protection, and control software	Technologically advanced and highly promising for precision automation	High initial cost, complex maintenance, and stringent safety requirements	High-value production systems, as well as research and demonstration applications	[58,60,65,70]
Safety control	Requires emission interlocks, emergency stop systems, human/animal detection, and anti-reflection design	Supports controlled and safe precision operation	Safety systems are complex, and risk control is difficult in open-field environments	Well-enclosed field robotic platforms	[60,63,70]

**Table 5:** Comparison of different technical routes for electrical weeding in terms of operating mechanism, advantages, limitations, application scenarios, and representative references.

Technical Route	Operating Mechanism	Main Advantages	Main Limitations	Typical Application Scenarios	Representative References
Continuous electrode-contact electrical weeding	Electrical current is delivered into the aboveground plant parts through direct electrode contact and then conducted toward the root system	Direct action; can affect both aboveground tissues and underground regenerative structures; no chemical residues	Strongly influenced by contact quality, crop occlusion, plant water content, and soil conductivity	Field conditions in which targets are relatively accessible, such as inter-row or near-crop zones	[73,75,78]
High-voltage arc/discharge electrical weeding	Applies high-energy impact to the target through instantaneous electrical discharge	Strong potential for non-contact treatment; theoretically rapid action	Requires high stability and stringent safety control; substantial risks of fire and unintended discharge	Specific equipment platforms or experimental research settings	[73,78]
Low-energy electrophysiological treatment (low-energy electrocution/microshock)	Uses low-energy, short-duration electrical stimulation to disrupt tissue function	Potentially high energy efficiency; better suited to small targets and robotic site-specific applications	Strong species-dependent variation; narrow parameter window; highly sensitive to treatment location	Seedling-stage weeds and precise intra-row treatment	[74,79,80,81]
Continuous inter-row electrical treatment	Continuously treats taller weeds in the inter-row space through direct electrical contact	Suitable for weeds protruding above the crop canopy or clearly exposed inter-row targets; relatively simple operational organization	Difficult to achieve plant-level selectivity; limited capacity for crop protection in near-crop zones	Orchards, vineyards, and cropping systems with relatively open inter-row space	[75,78,82]
Plant-specific robotic electrical weeding	Combines vision-based recognition with targeted electrode contact for individual weed treatment	May reduce energy consumption per unit area and minimize non-target effects; well suited to integration with AI-based perception	Depends on high-accuracy recognition, localization, and end-effector control; system integration is complex	High-value crops, precision agriculture, and research-oriented robotic platforms	[73,79,82]
Repeated electrical treatment strategy	Suppresses perennial or regenerative weeds through multiple rounds of treatment	Greater potential against persistent weeds and underground regenerative structures	Increases operating cost and time input; economic trade-offs must be considered	Organic agriculture and perennial weed management	[73,78,82]

**Table 6:** Comparison of other emerging weeding approaches in terms of mechanism, advantages, limitations, development stage, and representative references.

Technical Route	Mode of Action	Main Advantages	Main Limitations	Current Development Stage	Representative References
Microwave weeding	Uses microwave dielectric heating to act on weed tissues or the shallow soil seed bank	Offers potential for volumetric heating and can target both aboveground tissues and shallow subsurface propagules	High energy consumption, high power requirements, and limited field operating speed	Experimental research and localized prototype exploration	[83,84]
Bioherbicides	Uses microorganisms, natural metabolites, or plant-derived bioactive compounds to suppress weeds	Strong potential for environmentally sustainable weed control and good compatibility with precision application	Limited field stability, difficulty in commercialization, and highly variable control efficacy	Ongoing research and limited commercialization efforts	[85,86]
Cold plasma weeding	Suppresses seed germination or seedling growth through reactive species, electrothermal effects, and surface damage	No chemical residues and strong potential as an advanced non-chemical technology	Insufficient field-scale validation and device stability; limited data on energy efficiency and treatment consistency	Early exploratory stage	[87]

**Table 7:** Representative quantitative performance indicators reported for different robotic weeding modalities.

Modality	Representative System or Study	Working Scenario	Operating Speed/Field Capacity	Energy or Input Dose	Positioning Accuracy/Targeting Requirement	Weed Control Efficacy	Crop Injury/Limitation	References
Mechanical intra-row weeding	Commercial Robovator intelligent cultivator	Broccoli and transplanted lettuce	Tractor-mounted intra-row cultivation; performance varied with weed density and crop spacing	No chemical input; mechanical actuation energy not separately reported	Requires accurate crop-row and crop-position detection for synchronized blade actuation	Removed 18–41% more weeds under moderate to high weed densities than a standard cultivator	Did not reduce crop stand or marketable yield compared with the standard cultivator; reduced hand-weeding time by 20–45%	[121]
Mechanical intra-row weeding	Electric swing-type opening–closing weeding device	Open-field cabbage	0.1–0.5 m s^−1^ tested	Electric actuation; servo motor rated power 750 W	Crop localization and dynamic crop avoidance required	Laboratory weeding accuracy reached 96.67% at 0.1 m s^−1^ and decreased at higher speed; field accuracy decreased to 81.79% at 0.5 m s^−1^	Crop injury increased from 0.83% at 0.1 m s^−1^ to approximately 5.49% at 0.5 m s^−1^	[36]
Chemical spot spraying	Deep-learning-based robotic spot sprayer	Sugarcane field trials	Tested over 25 ha of field trials	Herbicide use reduced by 35% on average and up to 65% in low-weed-pressure strips	Requires nozzle-level spatial–temporal synchronization between detection and spraying	Achieved weed control equivalent to 97% of broadcast spraying	Still depends on herbicide susceptibility and spray deposition; performance varies with weed density	[48]
Thermal hot foam	Hot-foam dose–response experiments	Non-crop or localized thermal treatment	Localized application; field-scale speed depends on machine configuration	3.33–8.33 L m^2^ hot foam effective depending on weed species; 6.67–8.33 L m^2^ delayed regrowth more strongly	Requires coverage of aboveground tissues rather than plant-level targeting	Several treatments achieved 100% weed cover devitalization within three days after treatment	Regrowth depends on weed species and dose; high water/heat demand remains a limitation	[103]
Thermal hot foam vs. hot water	Comparative hot foam and hot water treatment	Synthetic-herbicide-free systems and urban/organic settings	Same machine used for hot water and hot foam treatments	Foam reduced required water dose by at least 2.5-fold compared with hot water alone	Requires sufficient surface coverage and heat residence	Difficult weeds died after lower doses of hot foam than hot water	Foam improves heat retention but still requires heated water and additional foaming equipment	[122]
Laser weeding	50 W thulium-doped fiber laser	Seedling-stage grass and dicot weeds	Laboratory dose–response; intended for autonomous laser platform integration	0.4–12.7 J mm^2^, approximately 1.3–39.9 J per 2 mm laser spot	Requires millimeter-level targeting of apical meristem or stem base	Highest efficacy at one-leaf stage for grass weeds and cotyledon stage for dicot weeds	Regrowth common at four-leaf stage; throughput decreases with weed density and target number	[61]
Electrical microshock weeding	Pulsed microshock flat-plate electrode	Greenhouse seedling treatment	Site-specific treatment of five plants m^2^ considered in energy estimates	0.1–0.9 MJ ha^−1^ for 100% control of several broadleaf seedlings	Requires reliable electrode contact with target leaves or plant–soil interface	Several broadleaf weeds were successfully controlled; grass weeds were less susceptible	Strong species dependence; electrode placement and soil moisture affect energy discharge	[80]
Electrical microshock weeding	Outdoor flat-plate electrode system	Field seedling treatment	Field treatment of five weeds m^2^	15 kJ ha^−1^ for some broadleaf species; 363–555 kJ ha^−1^ for in-ground Lolium multiflorum depending on electrode placement	Requires contact or close interaction between electrode and weed tissue	Better than 90% control achieved for all tested species	Energy requirement increased when plants were pressed to soil, especially under wetter soil/contact conditions	[123]

## Data Availability

No new data were created or analyzed in this study. Data sharing is not applicable to this article.
